# Using the Social–Ecological Model to Assess Vaccine Hesitancy and Refusal in a Highly Religious Lower–Middle-Income Country

**DOI:** 10.3390/ijerph21101335

**Published:** 2024-10-09

**Authors:** Rachael M. Chait, Anindrya Nastiti, Delfi Adlina Chintana, Putri Nilam Sari, Nabila Marasabessy, Muhamad Iqbal Firdaus, Mila Dirgawati, Dwi Agustian, Heidi West, Herto Dwi Ariesyady, Tomoyuki Shibata

**Affiliations:** 1 Miller School of Medicine, University of Miami, Miami, FL 33136, USA; rmchait@med.miami.edu; 2 Global Environmental Health LAB, Los Angeles, CA 90034, USAherto@ftsl.itb.ac.id (H.D.A.); 3 Faculty of Civil and Environmental Engineering, Bandung Institute of Technology (ITB), Bandung 40132, West Java, Indonesianilam@ph.unand.ac.id (P.N.S.); nabilamrsy@gmail.com (N.M.); iqbalfirdaus015@gmail.com (M.I.F.); 4 Faculty of Medicine, Padjadjaran University (UNPAD), Jatinangor 45363, West Java, Indonesia; adlinadelfi@gmail.com; 5 Faculty of Civil Engineering and Planning, National Institute of Technology (ITENAS), Bandung 40124, West Java, Indonesia; mila.dirgawati@itenas.ac.id; 6 Department of Public Health, Faculty of Medicine, Padjadjaran University (UNPAD), Bandung 40161, West Java, Indonesia; 7 Department of Health Science, California State University, Long Beach, CA 90840, USA; 8 Public Health Program, College of Health and Human Sciences, Northern Illinois University, DeKalb, IL 60115, USA

**Keywords:** immunization, health promotion, vaccine hesitancy, vaccine refusal, lower middle-income county, religion, family dynamics, Indonesia, Social–Ecological Model

## Abstract

(1) Background: The aim of this study was to understand the factors associated with vaccine hesitancy and refusal in Indonesia using the Social–Ecological Model (SEM). (2) Methods: Data on demographics, religiosity, family dynamics, and perceptions of public health efforts were collected through an online survey and compared to the rates of vaccine hesitancy and refusal. (3) Results: Income and sex were significantly associated with vaccine hesitancy. Based on a vaccine passport policy to enter public spaces, people who felt inhibited to enter public spaces or perceived privacy threats were twice as likely to exhibit vaccine hesitancy. Participants who believed that religious groups had a difficult time getting vaccinated were nearly twice as likely to exhibit vaccine hesitancy and three times more likely to exhibit vaccine refusal. However, participants who believed in a higher religious power were 58% less likely to exhibit vaccine hesitancy. Religious leaders significantly influenced participants to make the decision regarding vaccination. Individuals with vaccine refusal were more than twice as likely to share information with others without fact-checking. Notably, structural barriers such as distance and transportation were most strongly associated with vaccine hesitancy and refusal. (4) Conclusion: Cultural factors play a significant role in vaccine hesitancy and refusal. The SEM can be used to propose multi-level interventions with collaboration and communication among stakeholders to improve community health.

## 1. Introduction

Vaccine hesitancy, characterized by a reluctance to receive vaccines, and vaccine refusal, defined as the decision to decline vaccination, rank among the top ten global health threats [1]. Vaccines currently prevent 4 million child deaths annually and have the potential to save an additional 50 million lives by 2030 [2]. Despite the increasing popularity of vaccines, various barriers have emerged, including structural, behavioral, and informational challenges [3]. Structural barriers involve issues like transportation and clinic availability; behavioral barriers include misperception and mistrust; and informational barriers encompass health literacy, cultural relevance, and misinformation [3].

Following the COVID-19 pandemic, vaccine hesitancy has continued to rise [4,5,6,7]. Parents who were hesitant about the COVID-19 vaccine and lacked trust in vaccination information now have controversial vaccine attitudes towards the recommended childhood immunizations for their children [8]. As the COVID-19 vaccine becomes routine, similar to the annual flu shot, implementing effective evidence-based interventions to address vaccine hesitancy is crucial. Previous studies on vaccine hesitancy have focused on knowledge and perceptions of vaccine safety to mitigate informational and behavioral barriers at the individual level [9,10,11,12]. In high-income countries, safety concerns predominantly influence hesitancy, where most current intervention efforts are concentrated [13]. However, in lower–middle-income countries (LMICs), social factors, cultural dynamics, and religious beliefs exert more influence on vaccine hesitancy [13,14]. Religious beliefs were found to be the leading cause of vaccine hesitancy during a qualitative analysis of thirteen high-, middle-, and low-income countries [15].

Vaccine hesitancy associated with cultural and religious factors in Asia remains understudied [16,17,18,19]. Indonesia, the third largest country, with 275 million people, and a LMIC country in Southeast Asia, offers a unique cultural blend of Asian patriarchal customs and Muslim family values [20]. Overall, 99% of the Indonesian population self-identifies as religious [21]. With 87% of its population identifying as Muslim, Indonesia represents the world’s largest Muslim population, accounting for 13% of global Muslims [21,22]. This unique culture is unparalleled compared to any other country. Only one known study documented Indonesians’ cultural misperceptions regarding COVID-19, showing how some Indonesians thought they were safe from COVID-19 due to its tropical climate and the belief that herbal medicines can cure COVID-19 [23]. In such contexts, religious beliefs can deeply influence public health behavior by supporting or contradicting scientific recommendations. Moreover, understanding factors associated with family and religion in Indonesia could inform culturally competent interventions addressing vaccine hesitancy at interpersonal and community levels in other LMICs. Social and religious concerns could be used as a leverage to enhance public health strategies and vaccine uptakes in populations that might otherwise be resistant.

Policy is crucial for ensuring vaccine uptake [24]. During the pandemic, most countries recommended citizens get the COVID-19 vaccine, but only a few countries, including Indonesia, implemented vaccine mandates [25]. Indonesia’s Ministry of Health introduced PeduliLindungi smartphone application, which issued digital vaccine certificates. Individuals were required to present or scan the QR code to enter public places such as stores, restaurants, workplaces, and schools. While PeduliLindungi could theoretically address vaccine hesitancy by encouraging vaccination, its impact on hesitancy and refusal has not been investigated. In Indonesia, examining the impact of a mandatory health app on vaccine decisions can illuminate how digital enforcement mechanisms interact with social values and religious beliefs, providing a holistic understanding of both technological and socio-cultural factors in public health interventions. This new insight will be highly relevant currently as the Indonesian Government decided to strengthen the digital health intervention by transforming the PeduliLindungi, which was mainly developed for COVID-19 control and prevention, into SatuSehat Platform, which is designed as an app to provide a digital transformation of health service delivery for all relevant diseases or health problems.

This study aimed to identify factors at multiple levels associated with vaccine hesitancy and refusal based on the Social–Ecological Model (SEM) to improve community health through the promotion of prevention in Indonesia. This model can be employed to adopt a multi-level approach, an aspect that has been underrepresented in the recent literature concerning vaccine hesitancy and refusal [4,6,11]. The SEM has been shown to capitalize on an interplay of factors on the societal, community, interpersonal, and individual levels, creating more sustainable solutions for populations [26]. Thus, this research sought to address knowledge gaps in developing culturally competent, system-based approaches for vaccine strategies and policies in LMICs. The findings will inform multisectoral stakeholder collaborations to enhance future vaccination efforts.

## 2. Materials and Methods

### 2.1. Study Site

This study took place in Bandung, West Java, Indonesia. Bandung, the capital city of West Java Province, has a population of 2.44 million.

### 2.2. Survey Design

This was a cross-sectional survey study. An Indonesia–US student–professor team collaboratively developed the questionnaire in English based on a literature review, which was then translated into Bahasa Indonesia by the Indonesian team to ensure accuracy. The survey was structured to examine the interplay of factors across multiple levels of the Social–Ecological Model, encompassing individual, interpersonal, community, and societal influences on vaccine-related behaviors and outcomes [26]. The questionnaire comprised nine sections: (1) Demographics; (2) COVID-19 Decision Making [27]; (3) Family Power Dynamics [28,29,30]; (4) Sources of COVID-19 and Vaccine Information [31]; (5) COVID-19 Vaccine and Perception [32]; (6) Vaccine Hesitancy Scale [33,34] (7) Religiosity Assessment [35]; (8) Religious Influence on Public Health [36,37]; and (9) Policy Intervention.

### 2.3. Variables and Measures

This study defined vaccine hesitancy as any reluctance in receiving a vaccine and defined vaccine refusal as any time a person refused a vaccine. Questions about vaccine hesitancy and refusal were asked in the context of any vaccine a person has received, not just specifically for the COVID-19 vaccine. Types of vaccine hesitancy were defined through the World Health Organization’s Strategic Advisory Group of Experts (SAGE) 5C model: confidence, complacency, constraints, calculation, and collective responsibility [38].

Information on the number of COVID-19 vaccine doses and information regarding whether a participant had ever tested positive was obtained. Participants were also asked about their sources of vaccine information and the frequency with which they verified this information using a Likert scale. The vaccine hesitancy scale was utilized to evaluate any hesitancy or refusal, concerns about side effects, and any existing misconceptions. The measures analyzed included prevalence of vaccine hesitancy and refusal and adjusted odds ratio.

The participants were asked about who influenced their decision-making processes in general, as well as specifically regarding vaccination and other health-related decisions (i.e., visiting a doctor, wearing a mask) using a Likert scale. Influencers including male heads of households, female heads of households, religious leaders, healthcare professionals, government entities, educational institutions and teachers, and friends were analyzed. Family dynamics were defined as patterns of interactions and roles among family members [39]. To assess family dynamics, the participants were asked about their family’s ability to compromise, communicate, resolve problems, and support each other.

Religiosity questions were based off a validated Likert scale using the Centrality of Religiosity Scale (CRS) [35]. The variables assessed included the extent of the participants’ belief in a higher power, the frequency of their prayer, and the significance of religion in their lives. Additional information was gathered to determine whether religion or their place of worship influenced or informed their health decisions.

In evaluating the impact of the PeduliLindungi mobile application, variables were analyzed based on the participants’ attitudes towards entering public spaces, their perception of increased safety due to the application, and concerns about potential privacy violations using a Likert scale.

### 2.4. Data Collection

The study protocol was approved by the ethical committees at the Institut Teknologi Bandung (Bandung Institute of Technology) (#KEP/II/2022/X/M200722AN/UVHI) and Northern Illinois University (IRB #HS16-0174). All data were self-reported, and the participants could skip any questions they felt uncomfortable answering, as outlined in the survey’s consent form. The questionnaire was digitized using KoboToolbox, a free online/offline data collection tool, and tested using a pilot study among 61 participants in July 2022 for clarity and cultural appropriateness before being sent to the field. For the final survey, a convenience sampling approach was used to recruit Indonesian residents aged 18 and older between March 2023 to October 2023. The survey was primarily distributed via email and social media in Java, with additional responses from Bali, Sumatra, and other major Indonesian islands.

### 2.5. Data Analysis

Data collected via KoboToolbox was organized in Microsoft Office 365 Excel. No individual data were excluded. In specific questions that had missing data, the missing data were excluded from statistical analysis. Likert scale responses were retrospectively converted to binomial variables for odds ratio calculations as needed. For instance, responses for agree and strongly agree were coded into “yes”, while responses for strongly disagree, disagree, and neutral were coded into “no”. Religiosity scores were calculated using the Centrality of Religiosity Scale (CRS), categorizing participants as religious (average score of 3–3.9) or highly religious (average score of 4+) [35].

Chi-square two-tailed test and *t*-test or median tests were used for categorical (i.e., nominal and ordinal) and numerical variables, respectively, to compare differences in demographics, religiosity, family dynamics, and perception of public health efforts between vaccine hesitancy and refusal. The crude odds ratio was used to evaluate associations between determinants of related variables and vaccine hesitancy. A logistic regression model was used to control for confounding variables, such as sex and residential setting (i.e., rural, urban), to calculate an adjusted odds ratio. The statistical significance level was set to 0.05 for detecting differences and associations. Analysis was conducted using IBM SPSS Statistics (Version 28) (IBM, Armonk, NY, USA).

## 3. Results

### 3.1. Study Population

This study had a total of 224 Indonesian participants who responded to the survey (Table 1). The average age of the study participants was 29.8, aligning with the median age of 31.5 in 2024 [21]. Most participants were from Bandung, West Java, with 79% living in urban areas and 21% in rural areas. There was a significant difference between sex and vaccine hesitancy (*p* = 0.042), with more males (52.6%) than females (46.2%) reporting hesitancy. When controlling for geographical setting, men exhibited a potentially higher likelihood of being hesitant compared to women (AOR = 1.7; 95% CI 0.968–2.95; *p* = 0.065), although this result may be influenced by the limited sample size (Table 8). The study participants were highly educated as most of them (82.1%) had a bachelor’s degree or higher. Compared to the general Indonesian population, 24.62%, 22.74%, and 30.22% above 15 years old finished elementary school, lower secondary school, and upper secondary school, respectively; only 10.15% finished college, indicating this sample was highly educated [40].

The study participants demonstrated a 100% COVID-19 vaccination rate, with 71.4% receiving three or more doses. Vaccine types administered included Sinovac (74%), AstraZeneca (35.2%), Pfizer (32.2%), Moderna (20.3%), and Sinopharm (8.4%). Despite such high vaccine coverage among the study participants, many of them (42.7%) had tested positive for COVID-19. Of those who tested positive, 33.3% tested positive before any vaccine, 35.4% tested positive after the first vaccine, and 31.3% tested positive after the second vaccine. Approximately one-third (34.4%) of participants reported experiencing vaccine hesitancy and 17.6% experiencing vaccine refusal at some point in their life, including before and during the pandemic. The vaccine hesitancy rate in this study aligns with the findings of previous studies in Indonesia, which found hesitancy ranging from 16 to 60% [23,41,42]. Individual and household incomes for participants who had experienced vaccine hesitancy showed approximately half and one million Indonesian Rupiah fewer, respectively, compared to those who did not experience vaccine hesitancy (*p* = 0.019 and 0.018). No significant differences in vaccine hesitation or refusal were observed based on other demographic factors, such as age, marital status, education, or number of children (*p* > 0.05).

### 3.2. Societal Factors

This study revealed mixed feelings about the vaccine certificate application, PeduliLindungi (Table 2). While almost half of participants (45.4%) considered its mandatory use ethical, 15.2% believed it did infringe on their privacy. Despite these concerns, most participants (92.9%) had installed the app on their devices. The app’s implementation appeared inconsistent, with participants reporting the need to show vaccine certificates only half of the time (50.7%) when entering public spaces. Contrary to its intended purpose of encouraging vaccine uptake and improving herd immunity, only 18.8% of all participants stated that the app influenced their public outing behaviors. Feeling that the phone application threatens privacy was the lowest scoring question and had a significant association with vaccine hesitancy (*p* = 0.037) and refusal (*p* = 0.003).

In terms of structural barriers, 18% of the study population felt that either distance, clinic timing, wait times, or cost inhibited them from getting a vaccine. Almost 80% of those who experienced structural barriers were from urban areas, and there was an equal number of males and females.

There was no significant association between app usage and likelihood of vaccine hesitancy or refusal. However, individuals who felt the app inhibited them from entering public spaces were more than twice as likely to demonstrate vaccine hesitancy (AOR = 2.27; 95% CI 1.10–4.67; *p* = 0.026) and refusal (AOR = 2.59; 95% CI 1.12–5.98; *p* = 0.026) when controlling for sex and geographical setting (Table 8). Notably, structural barriers beyond individual control, such as distance, clinic timing, and transportation issues, were most strongly associated with vaccine hesitancy (AOR = 4.06; 95% CI 1.75–9.43; *p* = 0.001) and refusal (AOR = 3.31; 95% CI 1.44–7.63; *p* = 0.005) when controlling for the same confounding variables (Table 8).

### 3.3. Community Factors

The religious composition of the participants was predominantly Muslim (81.5%), followed by Catholic (7.0%), Christian (6.6%), and Protestant (3.1%). This distribution closely mirrors the Indonesian population according to the latest census: 87.2% Muslim, 7.5% Protestant, and 3.1% Catholic [21]. Among Muslims, 84.8% identified as highly religious, while 15.2% identified as religious based on the religiosity test. For other religions, the majority were highly religious (Table 3). There was no significant correlation between high religiosity and vaccine hesitancy or refusal.

When controlling sex and geographical setting, participants who believed that religious groups had a difficult time getting vaccinated were nearly twice as likely to exhibit vaccine hesitancy (AOR = 2.82; 95% CI 1.53–5.21; *p* < 0.001) and three times more likely to exhibit refusal (AOR = 3.48; 95% CI 1.66–7.30; *p* = 0.002) (Table 8). Conversely, participants who believed a higher power supported the COVID-19 vaccine had a 58% lower likelihood of vaccine hesitancy (AOR = 0.415; 95% CI 0.217–0.792; *p* = 0.008) (Table 8). There was no significant evidence that praying to a higher power (*p* = 0.155) or concerns about the vaccine’s religious compliance (i.e., Halal) (*p* = 0.112) increased hesitancy or refusal (Table 4).

### 3.4. Interpersonal Factors

Most participants felt their families could communicate effectively, discuss and solve problems, and listen to each other. There was no significant difference between family dynamics and vaccine hesitancy (Table 5). Only 66.7% of those were hesitant and 67.5% of those who refused felt that people were willing to help in an emergency, which was the lowest scoring category of all the family dynamic questions.

In Indonesia, males traditionally serve as the head of the household [20]. In this study, 67.8% of participants identified a male head of household influence, while 61.7% identified a female head of household influence. However, there was no significant difference in vaccine hesitancy based on the influence of either male or female heads of household (Table 6). Interestingly, the male and female heads of household had equivalent quantifiable influence in both the hesitant and refusal groups regarding vaccine and other health-related decisions. Religious leaders were significantly associated with vaccine decisions in both the vaccine-hesitant (*p* = 0.027) and refusal participants (*p* = 0.005). School and teachers had a significant influence on other health-related decisions in the hesitant group (*p* = 0.032). Male head of households had a significant influence on vaccine decisions in the refusal group (*p* = 0.016).

When controlling for sex and geographical setting, Muslims demonstrated higher odds of being influenced by male heads of household regarding decisions to get vaccinated (AOR = 2.03; 95% CI 1.02–4.07; *p* = 0.042) (Table 8). Highly religious participants, based on the religiosity scale which analyzed both Muslims and the other religious respondents, exhibited notable distinction in family dynamics, particularly in their ability to maintain familial respect (AOR = 3.65; 95% CI 1.34–9.87; *p* = 0.011) (Table 8).

### 3.5. Individual Factors

Misperceptions and mistrust significantly increased the likelihood of vaccine hesitancy among participants. Hesitant and refusal participants trusted and used the same sources (Table 7). Among hesitant participants, healthcare providers were identified as the most trusted vaccine information source (34.6%), followed by the government (30.8%) and the internet (17.9%). Similar patterns followed in the refusal group. However, the sources most frequently used by hesitant participants differed from the most trusted: social media (37.2%), the internet (30.8%), and the government (10.3%). A small percentage overall (1.3%) trusted religious sources, but none used them for COVID-19 vaccine information. Over half of the participants (53.3%) felt that social media information about the COVID-19 vaccine was negative. Additionally, 11% of participants shared such information without fact-checking, raising concerns about the spread of misinformation.

When controlling for sex and geographic setting, those who had ever heard negative information about vaccines were almost four times as likely to be hesitant (AOR = 3.78; 95% CI 1.60–8.93; *p* = 0.002) and five times as likely to refuse (AOR = 5.17; 95% CI 1.43–18.7; *p* = 0.012) (Table 8). Individuals with vaccine refusal (AOR = 2.67, 95% CI 1.05–6.83 *p* = 0.040) were more than twice as likely to share information with others without fact-checking. Participants who were not hesitant were more than two times more likely to agree to get the booster (AOR = 0.428, 95% CI 0.213–0.863, *p* = 0.018), and participants who had never refused vaccines were 2.7 times more likely to agree to get the booster (AOR = 0.368, 95% CI 0.165–0.822, *p* = 0.015) (Table 8). These findings emphasize the importance of addressing misperceptions and improving access to accurate vaccine information to reduce vaccine hesitancy.

## 4. Discussion

### 4.1. Societal Intervention

Indonesia was one of the few countries that mandated the COVID-19 vaccines, thus mitigating the complacency and promoting the collective responsibility outlined in the WHO’s 5C model [25,38]. To implement this policy, the Indonesian government required individuals’ digital vaccine certificates, so called “vaccine passports”, to access public place. This created a unique opportunity to investigate the challenges and opportunities in mandatory vaccination policy. However, due to limited financial support and lack of enforcement, the infrastructural weakness allowed people to still travel to places without presenting vaccine certificates, validated by only half the participants presenting vaccine certificates when entering public places. Rural areas were also less likely to check vaccine certificates than urban settings. The implementation of Indonesia’s unique vaccine passport system fell short of the government’s efficacy expectations. Nonetheless, Indonesia made significant efforts to reduce virus transmission in public places, aiming to protect vulnerable populations, including those unable to receive vaccines for medical or religious reasons.

The ethics of a vaccine mandate remain complex. Almost all participants in this study (91.2%) had the vaccine passport application, yet half still thought this mandate was unethical. Consequences of vaccine mandates include threats to rights and freedoms and a decrease in public trust, which can increase reluctance to engage in public health interventions [24]. In fact, the WHO does not support vaccine mandates and instead encourages a focus on informational campaigns and vaccine access [24] To motivate the public to comply with the recommendations, governments are encouraged to collaborate with local stakeholders, including non-government organizations, industries, and academic institutions, that can endorse and advocate the importance of public health policies and promote confidence, improving the ethical frustrations [24]. Additionally, substantial improvements in infrastructure and compliance monitoring would be essential to enforce the policy. The lessons learned from Indonesia’s experience offer valuable insights for numerous countries, including the United States, to refine existing policies and the development of more robust, user-friendly systems for verifying vaccination status, ultimately contributing to more effective public health measures in the context of global mobility.

Constraints defined by the 5C model, such as distance to clinics and appointment timing, were the primary barriers to vaccine compliance in the Indonesian study site. This aligns with research from Myanmar, another Southeast Asian LMIC, which identified distance and transportation to healthcare facilities as significant risk factors for poor healthcare-seeking behaviors [43]. These results and others from the region underscore the critical need for governments and healthcare sectors to enhance accessibility by addressing social and economic determinants of health [14,44]. The pandemic-era practice of utilizing mosques and churches as community vaccination centers exemplifies an effective strategy to mitigate structural barriers [45]. Expanding religious collaborations could significantly reduce accessibility-related obstacles and improve vaccine uptake in future public health initiatives without having to mandate vaccination [15].

### 4.2. Community Intervention

Religion can be influential in vaccine hesitancy and refusal, promoting confidence outlined in the 5C model [38]. There is mixed evidence on the impact of religion on vaccine decisions [46,47]. Religion is acknowledged by the WHO Vaccine Hesitancy Matrix as a confounder [15]. Religious influence can also be confounded with socioeconomic factors within the religious group that influence vaccine hesitancy [47]. It is undeniable that religious leaders and groups can have significant influence on its followers. Religion was a protective factor when participants claimed they believe a higher power supported vaccines. Additionally, religious leaders were significantly influential among the participants who were highly religious.

Religion can promote values and service to protect health of others, which can include vaccination [45]. An important intervention on the community level would be to target religious leaders and faith-based organizations to partner with healthcare workers to provide factual information on vaccines for their communities. Religion has been used to help reduce the spread of infectious disease such as HIV, polio, and Ebola [48]. In Pakistan, Muslim religious leaders were used to campaign for the polio vaccine to combat misinformation [49]. In Malaysia, religious leaders were educated to help reduce the HIV stigma by preaching to their congregants on compassion for those with HIV and instructing on proper religious burials for those people [50]. One research study analyzed the use of Catholic priests in Mexico to start a diabetes program to help educate people in their community [51].

Faith-based organizations (FBOs) are effective in promoting vaccine acceptance globally, specifically targeting underserved and minority populations that are highly religious [52]. During the COVID-19 pandemic in Indonesia, the Central Board, or head governance structure of the FBOs, supported the COVID-19 vaccine and was able to partner with the government to educate and mobilize vaccine promotion efforts [53]. Indonesia has many rural areas with little state presence where religious organizations have become the main presence, and targeting the FBOs during the pandemic was the grassroots approach to promoting vaccine implementation [53]. Religion has a powerful network and communication level, specifically with the ability to reach out to rural and uneducated levels. Thus, utilizing religious leaders and the intersection with health promotion can have a positive influence on vaccine acceptance.

### 4.3. Interpersonal Intervention

Southeast Asian culture is characterized by a strong patriarchal structure, where the male head of household holds significant respect and influence [20]. Indonesia exhibits higher levels of discrimination against women, particularly within family structures, compared to other Southeast Asian countries and global averages [20].This disparity stems from historical precedents, such as the 1974 Law of Marriage designating males as household heads, and Sharia principles defining women’s roles as subservient to husbands and families [20]. To address these issues, multisectoral stakeholders must collaborate to raise awareness of gender inequalities and implement actions promoting gender equality, aligning with the Sustainable Development Goals (SDGs). Such efforts are crucial for fostering a more equitable society and improving overall health outcomes in Indonesia.

In this study setting, men demonstrated more hesitancy than women, which is not consistent with other studies that show women exhibit more vaccine hesitancy, especially when pregnant [54,55]. This result could be due to the fact that the female participants in this study were highly educated compared to the average population. There were no specific differences in family dynamics concerning vaccine hesitancy or refusal; however, Indonesian traditional cultural norms often position men as household decision-makers. These findings highlight the potential significance of empowering women, who could be less hesitant than men with upper education, to promote vaccine acceptance and mitigate hesitancy.

Educating and empowering female heads of households (i.e., mothers, grandmothers) to influence families on vaccine information could be a successful intervention on the interpersonal level to improve vaccine hesitancy. Doing so would improve confidence and trust in the healthcare system, which the 5C model outlines as a risk factor for hesitancy [38]. Women’s empowerment is the process of educating and informing women to have the agency to make their own choices [56]. Children whose mothers had higher levels of empowerment had better children health outcomes (height, weight, nutrition status) in sub-Saharan Africa [56]. An analysis across 50 LMICs found that women who had higher levels of empowerment had a higher incidence of children with vaccine completion for diphtheria, pertussis, and tetanus, and women with lower empowerment were three times more likely to have children with missing immunizations [57]. Additionally, a randomized control trial in Indonesia found that women more frequently served as local community health workers, and they were the only group that significantly improved vaccination registration rates in local villages [58]. Woman’s agency within households is known to be associated with vaccine uptake [59]. By empowering female heads of households, mothers and grandmothers can act as key decision-makers in regard to health within families [60]. Empowerment programs could teach women to confidently discuss vaccine benefits, address misinformation, and make informed choices. Having local female healthcare workers or respected community figures lead these initiatives would further increase trust and receptiveness [56]. Moreover, equipping female heads of households with knowledge about vaccine schedules, appointment booking, and transportation options will decrease the structural barriers.

### 4.4. Individual Intervention

Informational barriers in vaccine hesitancy relate to health literacy, cultural relevance, and misinformation [3]. Vaccine hesitancy has been found to be directly related to misinformation [11,13,61]. One study found that within a 6-month span, 73% of participants reported being exposed to misinformation, which directly linked to hesitancy [62]. This study population followed similar patterns to previously proven behaviors: mistrust triggered by false information and misperceptions about vaccine efficacy and side effects. Information sharing from people who are hesitant or have refused may become sources of mis- and dis-information, potentially contributing to the informational barriers that lead to vaccine hesitancy [3]. The 5C model outlines that calculation, the process of weighing risk and benefits of vaccination, is a factor contribution to vaccine hesitancy which can be improved with accurate vaccine information [38].

Another informational barrier observed was the discrepancy between the most reliable source compared to the most used source for vaccine information. Receiving vaccine information from the internet and social media, which reflect the top sources used in this study’s participants, have been proven to increase vaccine hesitancy [63,64,65]. Religion was not found as a reliable or used source to find out about vaccine information in this study. There were no other studies found that linked religion as a source for vaccine information. This study also shows that newer generations do not try to seek reliable information but rather use whatever source is most convenient, suggesting that interventions to promote reliable sources for health education would be helpful. Additionally, a majority of this study’s participants (89%) responded that they were fact-checking before spreading information. Fact-checking has been proven to reduce misinformation, which may serve as a protective factor [66]. Receiving information from the trusted source of healthcare providers has been shown to improve vaccine rates among children [63].

To minimize informational barriers to accessing reliable sources, individuals should be provided with trustworthy facts via social media platforms to make factual information convenient. As many as 92.14% and 74.80% Indonesian aged 15–24 and 25–64 years old, respectively, own a mobile phone [67]. The average Indonesian person spends more than five hours a day on mobile apps [68]. Indonesians are the largest users of two major social media globally: Facebook and Twitter [41]. A study analyzing social medical usage in Jakarta in the Gen Z population during the pandemic found that the most used social media was Instagram due to the fact that it contains information in the forms of images and videos, which are easier to understand and access than information in text [69]. Bringing accessible and reliable information to social media could bridge the gap between the most used and most trusted sources. Research has shown the effectiveness of using social media in public health communication, especially to target younger populations [70]. However, social media may not be useful for older adults in the rural Indonesian community [70]. Overall, leveraging the widespread use of social media can help disseminate important public health information and reduce misinformation.

### 4.5. Limitations and Future Studies

Overall, this study provides a multifactorial approach to address vaccine hesitancy and refusal successfully. Interventions on each level of the SEM would facilitate more effective vaccine policy development and implementation. The limitations of this study include a small sample size, especially lacking participants from rural areas, which is a common challenge with online surveys that are distributed by academic institutions located in an urban setting. To collect voices from rural areas, scholars need to expand their networks and collaborations with local healthcare facilities, such as *Puskesmas* (*pusat kesehatan masyarakat* or government-operated community health clinics) and community health outreach volunteers, such as *posyandu cadres,* who can reach out to individuals even in rural settings. However, even though rural areas in West Java may have lower cumulative incidence of COVID-19, the percentage of the rural population that was vaccinated was comparable to that in urban areas, indicating that the lack of rural responses does not pose a large threat to validity [71]. Another limitation is the lack of access to health information on the participants, which can be an important aspect to understand vaccine hesitancy [14].

Additionally, despite using a validated vaccine hesitancy scale from the WHO, certain questions on the survey could potentially be vague to the participants regarding specific factors or may not have translated directly from English to Bahasa Indonesian. Many people also selected “other” in the survey, and there was no further information captured on what “other” meant. Future surveys should be more specific on what “other” entails. Future studies need to account for participants in rural settings and of older generations. Further understanding of cultural barriers impacting vaccine hesitancy and refusal can inform future providers and public health practitioners to provide culturally competent care. Randomized control studies on possible interventions could also contribute to develop effective ways to decrease vaccine hesitancy and refusal. The intersection of cultural barriers with socioeconomic status, health literacy, and education can be another opportunity to improve understanding.

## 5. Conclusions

This study shows how policy, religion, family influence, and accurate vaccine information play significant roles in vaccine hesitancy and refusal. This study found that while almost half of the participants considered the mandatory use of a national health app ethical, some of them felt it infringed upon privacy, which could lead to inconsistent app implementation. Concerns about privacy were significantly associated with vaccine hesitancy and refusal. Although high religiosity was not associated with hesitancy, belief in divine or religious support for vaccines reduced hesitancy, suggesting the importance of religious leaders and faith-based organizations to promote vaccines. Empowering women was also found to be an important influence on the family level to mitigate any hesitancy or refusal. Moreover, this study found that misperceptions and mistrust combined with negative vaccine information were significant drivers of hesitancy, highlighting the need for accurate information which can be improved by providing accurate information conveniently through social media.

This study emphasizes the importance of targeting interventions at the societal, community, interpersonal, and individual levels to understand cultural factors relating to vaccine hesitancy and refusal. Collaboration and communication among stakeholders of the different levels is necessary for interventions to improve community health, especially promoting primary prevention efforts such as vaccination. Lessons learned about vaccine hesitancy from this study will be used in improving current and future vaccine deployments.

## Figures and Tables

**Table 1:** Demographics associated with vaccine hesitancy and refusal.

	Overall	Hesitant	Refusal
	(*n* = 224)	(*n* = 78)	(*n* = 40)
	No (%)	No. (%)	No. (%)
Average Age	29.8 ± 10	28.9 ± 9.2	27.6 ± 7.8
Sex			
Male	100 (44.6)	41 (52.6) *^,b^	22 (55.0)
Female	124 (55.4)	36 (46.2) *^,b^	17 (42.5)
Marital Status			
Married	70 (31.3)	25 (32.1)	13 (32.5)
Single	150 (67.0)	51 (65.4)	25 (62.5)
Median Monthly Income			
Individual (IDR in Millions) ^a^	4.5	4.0 *	3.5 **
Household (IDR in Millions) ^a^	5.5	4.0 **	3.2 **
Religion			
Muslim	184 (82.1)	66 (84.6)	38 (95.0)
Christian	15 (6.7)	4 (5.1)	1 (2.5)
Catholic	15 (6.7)	4 (5.1)	1 (2.5)
Protestant	7 (3.1)	3 (3.8)	0 (0)
Buddhist	2 (0.9)	0 (0)	0 (0)
Hinduism	2 (0.9)	1 (1.3)	0 (0)
Living Area			
Rural	47 (21.0)	15 (19.2)	11 (27.5)
Urban	177 (79.0)	63 (80.8)	29 (72.5)
Children (<17 years old)			
With Children	53 (23.7)	18 (23.0)	10 (25.0)
Without Children	172 (76.8)	60 (76.9)	30 (75.0)
Number of People in Household	3.7 ± 1.7	3.9 ± 1.8	3.5 ± 1.9
Occupation			
Student	50 (22.3)	22 (28.2)	5 (12.5)
Education	32 (14.3)	10 (12.8)	5 (12.5)
Industry, Trade and Services	25 (11.2)	11 (14.1)	9 (22.5)
Health	19 (8.5)	7 (8.9)	3 (7.5)
Water, Sanitation, and Waste Management	15 (6.7)	4 (5.1)	4 (10.0)
Energy and Extractives	12 (5.4)	4 (5.1)	0 (0)
Other	35 (15.6)	12 (15.3)	8 (20.0)
Highest Education			
University (Bachelor’s Degree or Higher)	184 (82.1)	63 (80.8)	30 (75.0)
Upper Secondary School (Grade 10–12)	13 (5.8)	13 (16.7)	8 (20.0)

Data for this table come from a community survey primarily distributed in Java, Indonesia. Due to the option for participants to skip questions they preferred not to answer, not all variables have a complete set of 224 responses (*n* = 224). A *t*-test, median test, or chi-squared tests, with a p-value threshold of less than 0.05, were used to evaluate statistical significance. ^a^ Monthly income is reported in Indonesian Rupiah (IDR). One million Indonesian Rupiah is equivalent to USD 64.07. * *p* < 0.05, ** *p* < 0.01 ^b^ One-sided *t*-test was used to assess sex with hesitancy and refusal.

**Table 2:** Perceptions of PeduliLindungi application and structural barriers associated with vaccine hesitancy and refusal.

	Overall	Hesitant	Refusal
	*n* = 224	*n* = 78	*n* = 40
	No (%)	No. (%)	No. (%)
Phone Application Usage	208 (92.9)	69 (88.5)	36 (90.0)
Application Perception			
Helpful in COVID-19 prevention	137 (61.2)	48 (61.5)	23 (57.5)
Feel safer going to public places	132 (58.9)	43 (55.1)	23 (57.5)
Inhibits entering public spaces	42 (18.8)	22 (28.2) *	14 (35.0) **
Threatens privacy	34 (15.2)	17 (21.8) *	12 (30.0) **
Believe it is ethical	102 (45.4)	39 (50.0)	17 (42.5)
Believe it is overall beneficial	149 (66.5)	50 (64.1)	24 (60.0)
Structural Barriers			
Inhibited by distance, timing, distance, wait times, or costs	40 (17.9)	28 (35.9) **	16 (40.0) **

Data for this table come from a community survey primarily distributed in Java, Indonesia. Statistical significance is based on the chi-square test between hesitant/non-hesitant and refusal/non-refusal with *p* < 0.05. * *p* < 0.05, ** *p* < 0.01.

**Table 3:** Religiosity by religion.

Religiosity Scale	Muslim	Christian	Catholic	Protestant	Buddhist	Hinduism
	(*n* = 184)	(*n* = 15)	(*n* = 15)	(*n* = 7)	(*n* = 2)	(*n* = 2)
	No. (%)	No. (%)	No. (%)	No. (%)	No. (%)	No. (%)
Highly Religious	156 (84.8)	14 (93.3)	10 (66.7)	6 (85.7)	1 (50.0)	2 (100)
Religious	28 (15.2)	1 (6.7)	5 (33.3)	1 (14.3)	1 (50.0)	-

Data for this table come from a community survey primarily distributed in Java, Indonesia. Religiosity scores were calculated using the Centrality of Religiosity Scale (CRS), categorizing participants as religious (average score of 3–3.9) or highly religious (average score of 4+) [35]. The CRS has been validated across multiple monotheistic and polytheistic religions [35]. All participants in this study fell into the category of religious or highly religious.

**Table 4:** Perceptions of religion and health associated with vaccine hesitancy and refusal.

	Overall	Hesitant	Refusal
	*n* = 224	*n* = 78	*n* = 40
	No. (%)	No. (%)	No. (%)
Pray for health to a higher power	208 (92.9)	75 (96.2)	38 (95.0)
Health is created and given by a higher power	209 (93.3)	73 (93.6)	37 (97.5)
Vaccines are a part of the health created and given by higher power	171 (76.3)	54 (69.2)	26 (65.0)
A higher power would support people getting the COVID-19 vaccine	154 (68.8)	45 (57.7) *	26 (65.0)
Pray often to a higher power for health	203 (90.6)	67 (85.9)	33 (82.5)
Concerned vaccines do not follow Halal or reli- gious dietary standards	143 (63.8)	44 (56.4)	24 (60.0)
Religion educates on the importance of health	211 (94.2)	76 (97.4) *	39 (97.5)
Religion educates on the importance of vaccines	144 (64.3)	46 (60.0)	27 (67.5)
Place of worship influences decision to take care of health	168 (75.0)	66 (84.6) *	33 (82.5)
Place of worship influences decision to get a vac- cine	131 (58.5)	46 (60.0)	25 (62.5)
Religion has a positive benefit on health	208 (92.9)	76 (97.4)	39 (97.5)
Religion has a positive benefit on motivation to get a vaccine	148 (66.1)	51 (65.4)	26 (65.0)

Data for this table come from a community survey primarily distributed in Java, Indonesia. Higher power refers to Allah, God, Gods, etc. Statistical significance is based on the chi-square test between hesitant/non-hesitant and refusal/non-refusal with *p* < 0.05. * *p* < 0.05, ** *p* < 0.01.

**Table 5:** Factors within family dynamics associated with vaccine hesitancy and refusal.

	Overall	Hesitant	Refusal
	*n* = 224	*n* = 78	*n* = 40
	No (%)	No. (%)	No. (%)
My family			
Compromises when problems come up	188 (83.9)	66 (84.6)	35 (87.5)
Talks about the way we communicate in our family	185 (82.6)	64 (82.1)	36 (90.0)
Consults with each other about decisions	193 (86.2)	68 (87.2)	35 (87.5)
Defines problems positively to solve them	183 (81.7)	65 (83.3)	35 (87.5)
Discusses problems and feel good about the solutions	181 (80.8)	64 (82.1)	33 (82.5)
Discusses things until we reach a resolution	182 (81.3)	65 (83.3)	36 (90.0)
Respects each other	192 (85.7)	70 (89.7)	36 (90.0)
Often listens to family members’ concerns or problems	176 (78.6)	63 (80.8)	35 (87.5)
Solves major problems	191 (85.3)	66 (84.6)	35 (87.5)
Accepts stressful events as part of life	180 (80.4)	65 (83.3)	35 (87.5)
Accepts that problems occur unexpectedly	183 (81.7)	66 (84.6)	35 (87.5)
Feels people are willing to help in an emergency	159 (71.0)	52 (66.7)	27 (67.5)
Knows there is a community to help if there is trouble	138 (61.6)	49 (62.8)	29 (72.5)
Attends church/synagogue/mosque services	196 (87.5)	69 (88.5)	35 (87.5)
Participates in religious activities	196 (87.5)	69 (84.6)	36 (90.0)

Data for this table come from a community survey primarily distributed in Java, Indonesia. Statistical significance is based on the chi-square test between hesitant/non-hesitant and refusal/non-refusal with *p* < 0.05. * *p* < 0.05, ** *p* < 0.01.

**Table 6:** Decision influences associated with vaccine hesitancy and refusal.

	Overall	Hesitant	Refusal
	*n* = 224	*n* = 78	*n* = 40
	No (%)	No. (%)	No. (%)
Vaccine Decision Influencers			
Male Head of Household ^a^	142 (63.3)	54 (69.2)	32 (80.0) *
Female Head of Household ^b^	127 (56.7)	49 (62.8)	26 (65.0)
Religion/Religious Leader	107 (47.8)	45 (57.7) *	27 (67.5) **
Healthcare Worker	184 (82.1)	68 (87.2)	34 (85.0)
Government	168 (75.9)	61 (78.2)	31 (79.5)
School/Teachers	118 (52.7)	47 (60.3)	23 (57.5)
Friends	91 (40.6)	33 (42.3)	20 (50.0)
Other Health-Related Decision Influencers ^c^			
Male Head of Household ^a^	153 (68.3)	59 (75.6)	32 (80.0)
Female Head of Household ^b^	161 (71.9)	62 (79.5)	32 (80.0)
Religion/Religious Leader	121 (54.0)	46 (59.0)	27 (67.5)
Healthcare Worker	185 (82.6)	68 (87.2)	33 (82.5)
Government	177 (79.0)	63 (80.8)	31 (77.5)
School/Teachers	136 (60.7)	55 (70.5) *	29 (72.5)
Friends	115 (51.3)	44 (56.4)	23 (57.5)

Data for this table come from a community survey primarily distributed in Java, Indonesia. Statistical significance is based on the chi-square test between hesitant/non-hesitant and refusal/non-refusal with *p* < 0.05. * *p* < 0.05, ** *p* < 0.01 ^a^ Father, husband, and grandfather. ^b^ Mother, wife, and grandmother. ^c^ Hand washing, wearing a mask, going to see a doctor, etc.

**Table 7:** Most trusted and most used sources associated with vaccine hesitancy and refusal.

	Overall	Hesitant	Refusal
	*n* = 224	*n* = 78	*n* = 147
	No (%)	No. (%)	No. (%)
Most Trusted Source			
Healthcare Provider	85 (37.9)	27 (34.6)	13 (32.5)
Government	57 (25.4)	24 (30.8)	11 (27.5)
Internet	43 (19.2)	14 (17.9)	5 (12.5)
Social Media	20 (8.9)	4 (5.1)	5 (12.5)
TV	7 (3.1)	3 (3.8)	3 (7.5)
Religion	3 (1.3)	2 (2.6)	2 (5.0)
Friends or Family	2 (0.9)	1 (1.3)	0 (0)
School/University	1 (0.4)	0 (0)	0 (0)
Most Used Source			
Social Media	86 (38.4)	29 (37.2)	15 (37.5)
Internet	61 (27.2)	24 (30.8)	11 (27.5)
Healthcare Provider	26 (11.6)	6 (7.7)	4 (10.0)
Government	24 (10.7)	8 (10.3)	4 (10.0)
TV	15 (6.7)	7 (9.0)	4 (10.0)
Workplace	6 (2.8)	0 (0)	1 (2.5)
Friends or Family	5 (2.2)	2 (2.6)	1 (2.5)
Religion	0 (0)	0 (0)	0 (0)

Data for this table come from a community survey primarily distributed in Java, Indonesia. Statistical significance is based on the chi-square test between hesitant/non-hesitant and refusal/non-refusal with *p* < 0.05. * *p* < 0.05, ** *p* < 0.01.

**Table 8:** Significant factors associated with vaccine hesitancy and refusal.

	Hesitant	Refusal
	AOR (95% CI)	AOR (95% CI)
Societal Factors		
Application (PeduliLindungi) inhibited entering public spaces	2.27 (1.10–4.67) *	2.59 (1.12–5.98) *
Inhibited by distance, timing, transportation, wait, and cost	4.06 (1.75–9.43) **	3.31 (1.44–7.63) **
Community (Religious) Factors		
Vaccination difficult for community religious groups	2.82 (1.53–5.21) **	3.48 (1.66–7.30) **
Higher power supports people getting the COVID-19 vaccine	0.415 (0.217–0.792) **	-
Individual Factors		
Identifying as male	1.7 (0.968–2.95) ^a^	-
Ever received or heard negative information about vaccines	3.78 (1.60–8.93) **	5.17 (1.43–18.70) *
Willingness to get recommended vaccine booster shots by the government	0.428 (0.213–0.863) *	0.368 (0.165–0.822) *

Data for this table come from a community survey primarily distributed in Java, Indonesia. Statistical significance is based on the chi-square test between hesitant/non-hesitant and refusal/non-refusal with *p* < 0.05. Participants who answered “yes” to the factor in the first column are more likely to experience vaccine hesitancy or refusal based on the adjusted odds ratio portrayed. Blank cells indicate that the factor was not significant when compared to vaccine hesitance or refusal with *p* > 0.05. * *p*< 0.05, ** *p* < 0.01. ^a^ *p*-value was 0.065 when controlling for residential settings (i.e., rural, urban).

## Data Availability

The raw data supporting the conclusions of this article will be made available by the authors on request.
